# Digital marking versus manual marking technique for toric intraocular lens alignment: a meta-analysis

**DOI:** 10.1186/s12886-025-04329-9

**Published:** 2025-09-02

**Authors:** Ji-guo Yu, Ting Ye, Yi Xiang

**Affiliations:** 1 Department of Ophthalmology, Tongji Medical College, The Central Hospital of Wuhan, Huazhong University of Science and Technology, No 26 Shengli Street, Wuhan, 430014 China; 2 Key Laboratory for Molecular Diagnosis of Hubei Province, Tongji Medical College, The Central Hospital of Wuhan, Huazhong University of Science and Technology, Wuhan, 430014 China; 3 Department of Ophthalmology, The Fourth Hospital of Wuhan, Wuhan, 430030 China

**Keywords:** Digital marking, Manual marking, Toric intraocular lens, Meta-analysis

## Abstract

**Background:**

Manual and digital-assisted marking are the two main marking methods used for toric intraocular lens (IOL) in cataract surgery. However, there is a lack of consensus regarding the consistency in accuracy for these two methods. This meta-analysis aimed to compare and evaluate the accuracy of digital and manual marking techniques for toric IOL alignment during cataract surgery.

**Methods:**

A comprehensive search was conducted using PubMed, Embase, and Cochrane Library databases to identify studies that compared the effectiveness of these two techniques in positioning toric IOLs. The primary outcome measures included postoperative uncorrected distance visual acuity (UDVA), corrected distance visual acuity (CDVA), mean deviation from target-induced astigmatism, and postoperative IOL misalignment. Statistical analyses were performed using RevMan 5.3 software.

**Results:**

This meta-analysis included four randomized controlled trials and three retrospective studies comprising 197 eyes in the digital marking group and 206 eyes in the manual marking group for comparative analyses. The statistical meta-analysis indicated no significant differences in postoperative UDVA (logMAR) between the two groups (*P* = 0.69). Additionally, the meta-analysis showed no significant differences in postoperative CDVA (logMAR) between the two groups (*P* = 0.29). However, the meta-analysis findings highlighted a significant reduction in the degree of postoperative IOL misalignment in the digital marking group compared to the manual marking group (*P* = 0.0001).

**Conclusions:**

The results of this meta-analysis offer compelling evidence that the digital marking technique resulted in a lower postoperative IOL misalignment rate than that for the traditional manual marking technique. Both marking methodologies were encouraged in clinical practice because of their effectiveness and accuracy; although, the digital marking technique was generally preferred and highly recommended.

**Trial registration:**

The protocol of this study was registered in PROSPERO (registration ID: CRD420251045237).

**Supplementary Information:**

The online version contains supplementary material available at 10.1186/s12886-025-04329-9.

## Background

A significant proportion of patients with cataracts suffer from astigmatism, more than one third of these patients experience astigmatism greater than 1.0 diopters [1, 2], a condition that adversely affects their visual function and overall quality of life [3]. To achieve high postoperative visual quality, the implantation of a toric intraocular lens (IOL) during cataract surgery remains an efficient and reliable approach for correcting corneal astigmatism [4]. Accurate alignment of the toric IOL axis with the intended target axis is a crucial step in the implantation process. Preoperative marking of the horizontal and astigmatic axis at the peripheral cornea is a commonly used method [5]. The manual marking technique involves creating slight scratches on peripheral cornea and subsequently staining it with dye. These scratches cause epithelial erosion, which reduces the accuracy of the markings. Additionally, staining after the scratches leaves a broad mark leading to few degrees of error [6]. Due to the development of an image-guided system, accurate astigmatism axis markers can be obtained during surgery without humans performing the corneal marking.

Currently, manual and digital-assisted marking are the two main marking methods used for toric IOL in cataract surgery. However, there is a lack of consensus regarding the consistency in accuracy for these two methods. In previous studies, the digital-assisted technique has been reported to be more accurate than the manual-marking technique for postoperative toric IOL alignment [7, 8]. Other studies have reported that the manual and digital marking techniques are equally effective and similar for toric IOL alignment [9, 10]. Given the conflicting opinions concerning this matter, we conducted a meta-analysis to comprehensively evaluate and compare the accuracy of manual and digital marking techniques for toric IOL implantation during cataract surgery.

## Methods

### Search strategy

This meta-analysis was performed in accordance with the Cochrane Handbook for Systematic Reviews of Interventions and the Preferred Reporting Items for Systematic Reviews and Meta-Analyses (PRSMA) Statement. The PRISMA 2020 Checklist is presented as Supplementary Material 1. Relevant clinical studies, published before March 1, 2025, were searched using the PubMed, Embase, and Cochrane Library databases. The search items were “manual marking,” “digital marking,” “image-guided system,” and “toric intraocular lens.” The search outcomes retrieved from various databases were imported into dedicated reference management software (EndNote X4; Omson Reuters, New York, NY, USA) to facilitate the identification and removal of duplicate articles. Subsequently, two authors (JGY and TY) independently screened the articles by reviewing their titles and abstracts and eliminated those that were deemed irrelevant. The remaining articles underwent a thorough full-text review to assess their eligibility based on the predefined inclusion criteria. Furthermore, the reference lists of the selected articles were scrutinized to identify additional relevant studies that might have been overlooked.

### Inclusion and exclusion criteria

Studies were included if they met the following criteria: (1) compared the digital and manual marking techniques; (2) evaluated toric IOL alignment in cataract surgery; (3) reported the required data including postoperative IOL misalignment, postoperative uncorrected visual acuity (UDVA), and corrected distance visual acuity (CDVA); and (4) follow-up duration was at least 3 months. The study exclusion criteria were as follows: (1) without a comparison group; (2) reported comparisons involving other marking technologies; (3) reported data could not be used for meta-analysis; (4) did not have follow-up or a follow-up duration less than 3 months; and (5) published as conference abstracts, research trials, case reports, or review articles. Two independent reviewers (JGY and TY) carefully evaluated the retrieved articles to identify those that met the inclusion criteria. Any disagreements encountered during this process were resolved through thorough discussions among the reviewers.

### Data extraction

Data from each study that met the inclusion criteria were independently extracted by two reviewers (JGY and TY). The following information was extracted: first author, publication year, study location, image-guided system, study design, number of eyes, mean age, sex, preoperative UDVA, preoperative astigmatism, and follow-up period. The main outcome parameters selected for the analysis included postoperative UDVA, postoperative CDVA, mean deviation from target-induced astigmatism, and postoperative IOL misalignment.

### Quality assessment

The potential for bias in the included randomized controlled trials (RCTs) was evaluated using the Cochrane risk of bias tool to ensure a rigorous and standardized assessment process [11]. Seven domains were assessed: (1) random sequence generation, (2) allocation concealment, (3) blinding of participants and personnel, (4) blinding of outcome assessment, (5) incomplete outcome data, (6) selective reporting, and (7) other biases. Each domain was classified as having a “low risk of bias,” “high risk of bias,” or “unclear risk of bias.” The methodological quality of the retrospective studies was assessed using the Newcastle-Ottawa Scale, which assesses three crucial elements: selection, comparability, and outcome or exposure. This scale assigns a score ranging from 0 to 9 points; studies that achieved scores ≥ 6 were deemed to have relatively high quality [12]. Two independent reviewers (JGY and TY) assessed the studies using these tools, and any discrepancies were resolved through discussions.

### Statistical analyses

Statistical analyses were performed using Review Manager 5.30 (Cochrane Collaboration, Oxford, UK). For continuous outcomes, the weighted mean difference (WMD) with a 95% confidence interval (CIs) was computed using the mean and standard deviation. To gauge the variability among the studies, the chi-squared test was employed, with *P* < 0.05 and I^2^ > 50% indicating substantial heterogeneity. When the I^2^ exceeded 50%, indicating high heterogeneity, a random-effects model was used for data analysis. Conversely, if I^2^ was ≤ 50%, indicating low to moderate heterogeneity, the fixed-effects model was utilized. A *P* value < 0.05 was deemed significant.

## Results

### Study selection

**Fig. 1:**
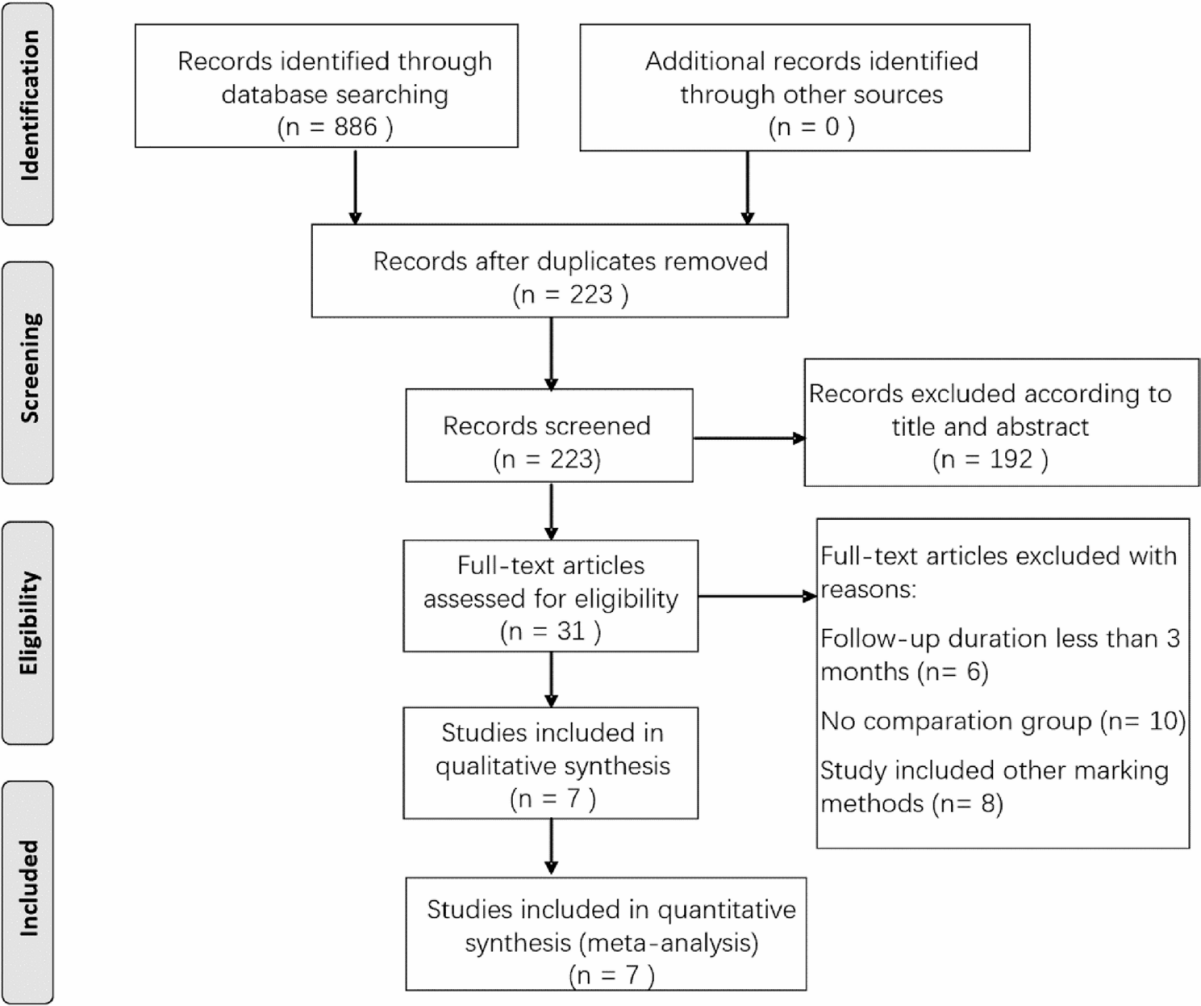
Flow chart of the literature search and selection

A total of 886 records were identified via electronic searches; 663 duplicate records were deleted, thus, 223 studies remained. Of these 223 studies, 192 were discarded after reviewing their titles and abstracts. The remaining 31 records were subjected to a comprehensive full-text review. Upon detailed examination, 10 articles were excluded due to the absence of any comparative analysis, six studies were dismissed because their follow-up periods were less than 3 months, and eight studies were omitted as they reported alternative marking methods. Ultimately, three retrospective studies and four RCTs that fulfilled our stringent inclusion criteria were included in the final meta-analysis [8–10, 13–16]. The study selection process is illustrated in Fig. 1.

### Characteristics of the included studies

**Fig. 2:**
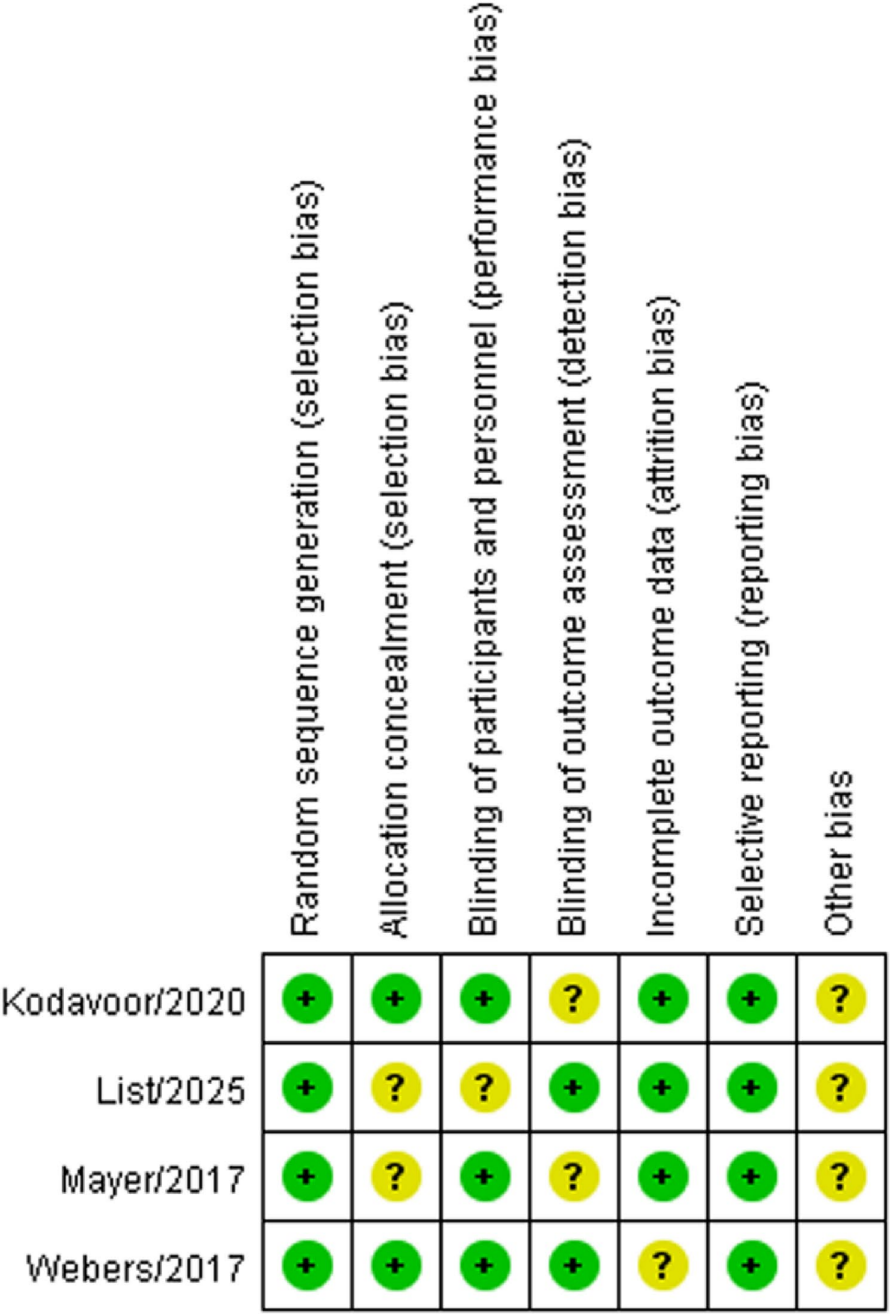
Risk of bias evaluation for the included randomized controlled trials. +, low risk of bias; -, high risk of bias;?, unclear risk of bias

The studies published between 2017 and 2025 comprised of 403 eyes, which were divided into two groups: 197 eyes in the digital marking group and 206 eyes in the manual marking group. Two studies were performed in Germany [14, 15], whereas the others were performed in China [13], Turkey [10], the Netherlands [8], Austria [16] and India [9]. Three studies were retrospective controlled studies [10, 13, 14] and four were RCTs [8, 9, 15, 16]. Patient ages ranged from 36 to 70 years and the number of eyes varied between 18 and 45. The average preoperative astigmatism ranged from 1.5 to 2.5 diopters, and the final follow-up time was 3 months for all patients. The fundamental features of each study are listed in Table 1. All three retrospective studies attained a Newcastle-Ottawa Scale score of seven stars, a clear indication of their high methodological quality and robustness (Table 2). Furthermore, the four RCTs incorporated in the analysis demonstrated a notably low risk of bias, ensuring the credibility and reliability of their findings (Fig. 2).

**Table 1:** Summary of the characteristics of the included studies

Author (Year)	Study location	System	Study design	No. of eyes		Mean Age (years)		Gender (Male/Female)		Preoperative UDVA (logMAR)		Preoperative astigmatism (D)		Follow up (month)
				DM	MM	DM	MM	DM	MM	DM	MM	DM	MM	
Kose/2020 [10]	Turkey	CES	Retro	45	35	62.2	62.3	19/26	15/20	0.64	0.65	2.26	2.37	3
Ding/2022 [13]	China	CES	Retro	36	36	62.72	60.17	11/17	8/22	0.82	0.72	1.98	2.21	3
Feldhaus/2023 [14]	Germany	CES	Retro	19	38	NR	NR	NR	NR	NR	NR	1.50	1.66	3
Mayer/2017 [15]	Germany	CES	RCT	29	28	NR	NR	NR	NR	0.63	0.70	2.20	2.40	3
List/2025 [16]	Austria	CES	RCT	20	20	36.3	36.3	4/16	4/16	NR	NR	1.8	1.82	3
Webers/2017 [8]	the Netherlands	Verion	RCT	18	18	68	70	9/9	12/6	NR	NR	2.15	2.47	3
Kodavoor/2020 [9]	India	Verion	RCT	30	31	62.68	63.04	14/11	14/11	0.54	0.63	1.72	2.01	3

*Retro* retrospective study, *RCT* randomized controlled trial, *CES* Callisto Eye System, *DM* digital marking, *MM* manual marking, *UDVA* Uncorrected distance visual acuity, *NR* not reported

**Table 2:** Newcastle–Ottawa scale results for the retrospective studies

Study	Year	Newcastle-Ottawa Scale			
		Selection	Comparability	Expose	Total Score
Feldhaus/2023 [14]	2023	**	**	***	7
Ding/2022 [13]	2022	**	**	***	7
Kose/2020 [10]	2020	**	**	***	7

The asterisk denotes the scoring results of items under the three crucial elements of the Newcastle–Ottawa scale

### Preoperative and postoperative UDVA (logMAR)

**Fig. 3:**
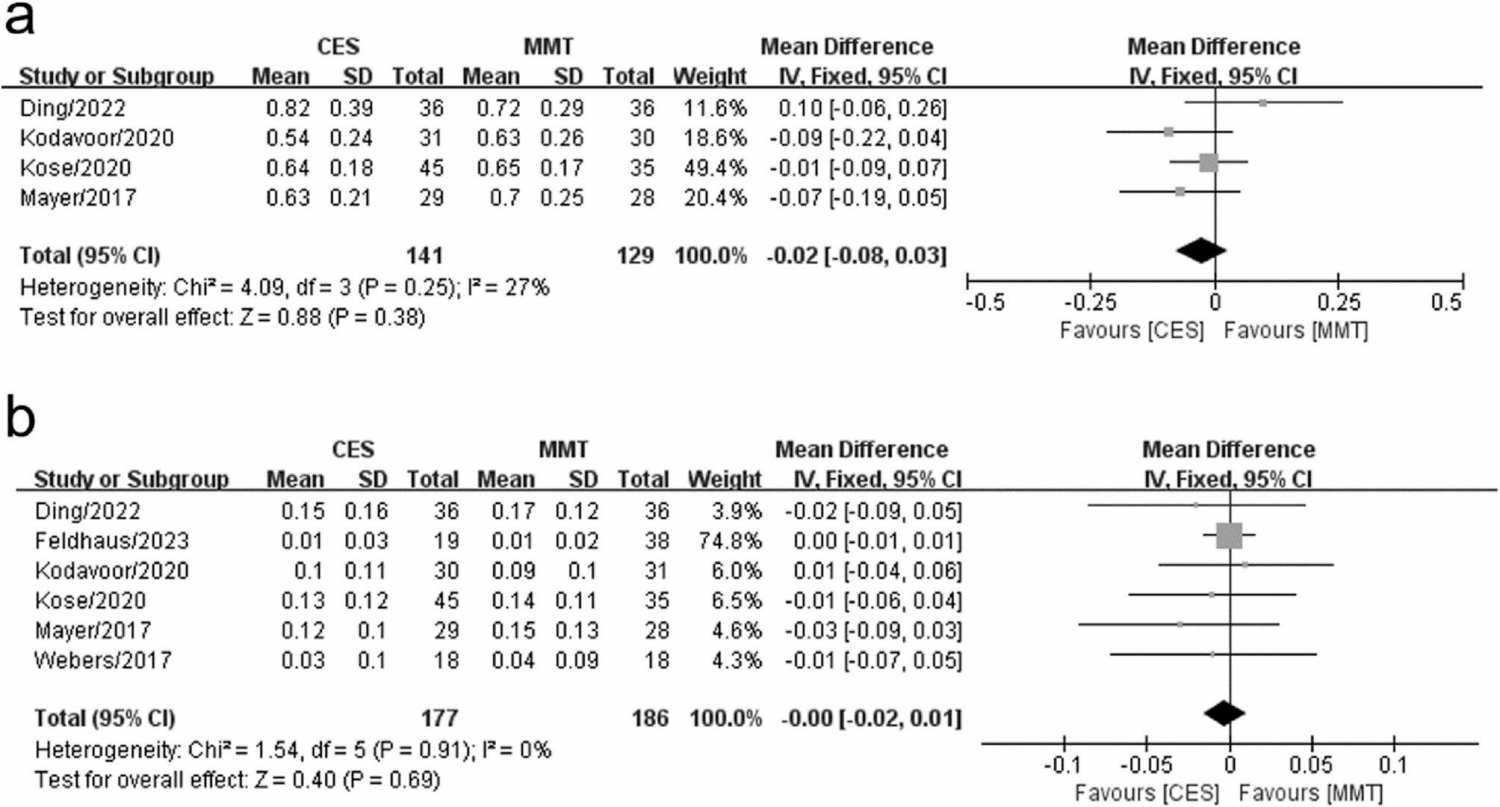
Forest plots of UDVA between the digital marking and manual marking groups (**a**) Preoperative UDVA (**b**) Postoperative UDVA

A comparison of preoperative UDVA (logMAR) between the digital and manual marking groups among four studies revealed no significant heterogeneity among the studies (I^2^ = 27%). Consequently, a fixed-effects model was employed for data analysis. The results of the meta-analysis indicated that the preoperative UDVA (logMAR) did not differ significantly between the two groups (WMD = −0.02, 95% CI: −0.08, 0.03, *P* = 0.38) (Fig. 3a).

The comparison of postoperative UDVA (logMAR) between the digital and manual marking groups among six studies at the 3-month follow-up revealed no significant heterogeneity (I^2^ = 0%). Consequently, a fixed-effects model was employed for data analysis. The meta-analysis indicated no significant difference in postoperative UDVA (logMAR) between the two groups. (WMD = −0.00, 95% CI: −0.02 to 0.01, *P* = 0.69) (Fig. 3b).

### Preoperative and postoperative CDVA (logMAR)

**Fig. 4:**
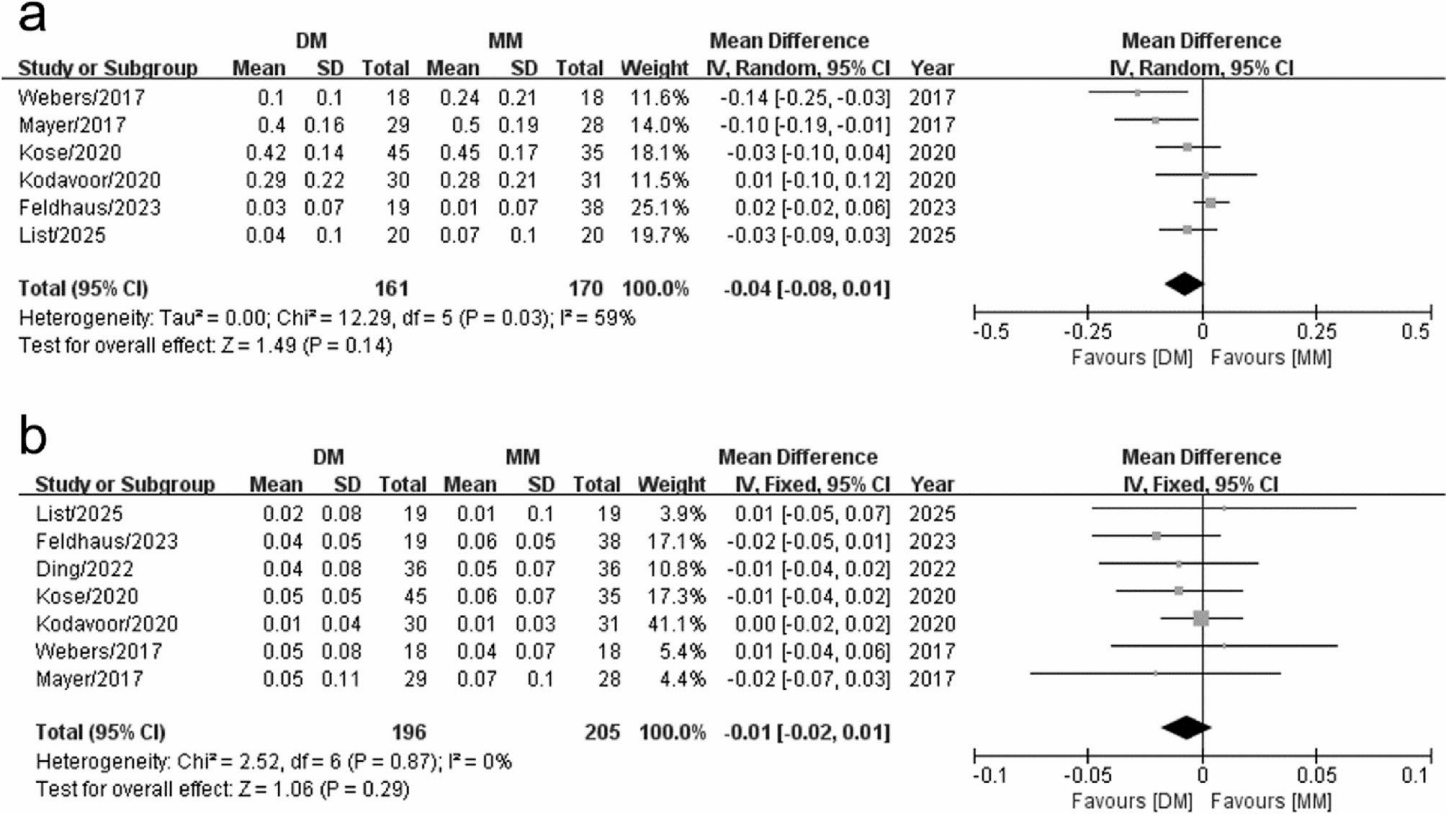
Forest plots of CDVA between the digital marking and manual marking groups (**a**) Preoperative CDVA (**b**) Postoperative CDVA

The preoperative CDVA (logMAR) was compared between the digital and manual marking groups among six studies, whic yielded significant heterogeneity in the results (I^2^ = 59%). Consequently, the data analysis employed a random-effects model. A subsequent meta-analysis revealed no significant difference in preoperative CDVA (logMAR) between the two groups. (WMD = −0.04, 95% CI: −0.08 to 0.01, *P* = 0.14) (Fig. 4a).

The postoperative CDVA (logMAR) was compared between the digital and manual marking groups at the 3-month follow-up among seven studies. No significant heterogeneity was found among them (I^2^ = 0%); therefore, the data were analyzed using a fixed-effects model. The meta-analysis of these data demonstrated that the postoperative CDVAs (logMAR) were not significantly different between the two groups. (WMD = −0.01, 95% CI: −0.02 to 0.01, *P* = 0.29) (Fig. 4b).

### Preoperative astigmatism

**Fig. 5:**
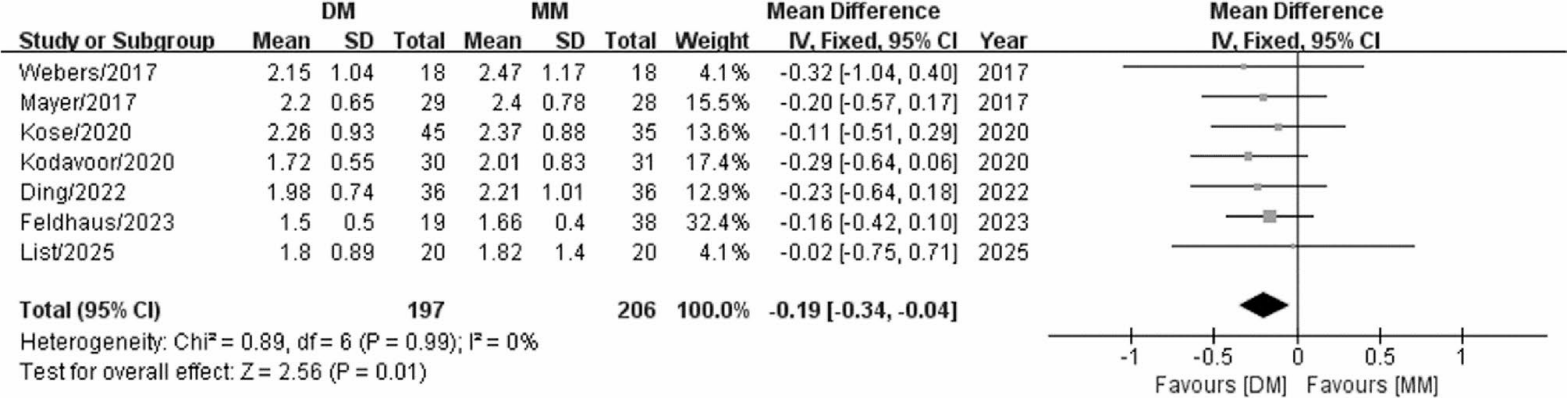
Forest plot of preoperative astigmatism between the digital marking and manual marking groups

Preoperative astigmatism was assessed in a comparison between the digital and manual marking groups among seven studies, which revealed no significant heterogeneity among the results (I^2^ = 0%). Consequently, the data were analyzed using a fixed-effects model. The meta-analysis of these data indicated that preoperative astigmatism was significantly higher in the digital marking group than in the manual marking group. (WMD = −0.19, 95% CI: −0.34 to −0.04, *P* = 0.01) (Fig. 5).

### Mean deviation from target-induced astigmatism (TIA)

**Fig. 6:**
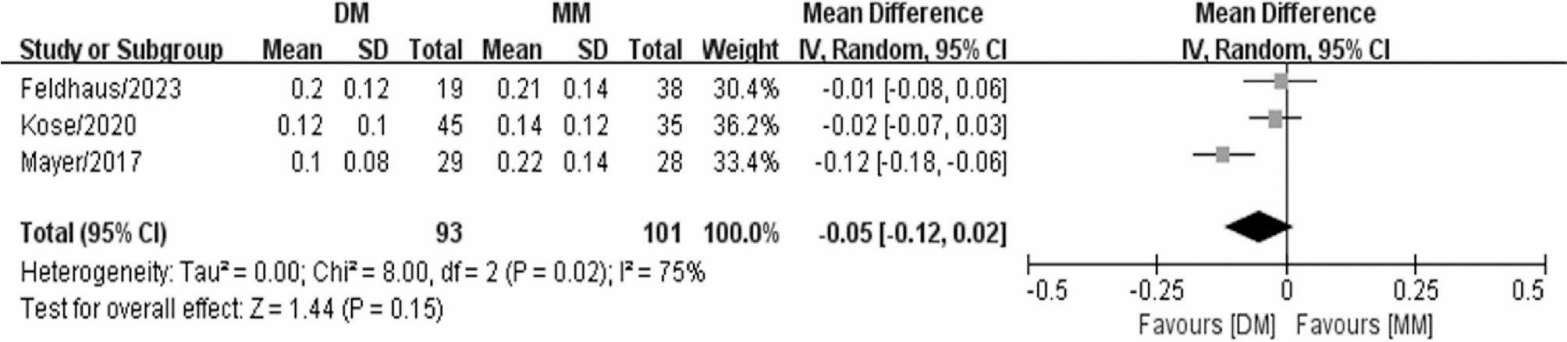
Forest plot of mean deviation from the TIA between the digital marking and manual marking groups

The mean deviation from the TIA was compared between the digital and manual marking groups among three studies. Significant heterogeneity (I^2^ = 75%) was found across the three studies; thus, the data were analyzed using a random-effects model. The meta-analysis of these data demonstrated that the mean deviation from the TIA was not significantly different between the two groups (WMD = −0.05, 95% CI: −0.12, 0.02, *P* = 0.15) (Fig. 6).

### Postoperative IOL misalignment (degree)

**Fig. 7:**
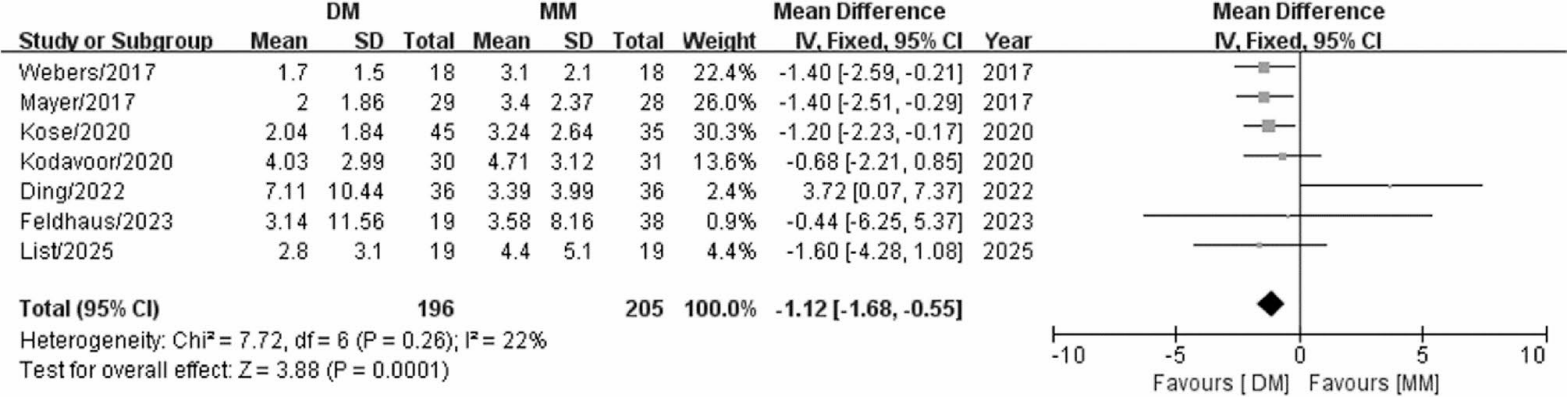
Forest plot of postoperative IOL misalignment (degree) between the digital marking and manual marking groups

The degree of postoperative IOL misalignment was compared between the digital and manual marking groups among seven studies. No significant heterogeneity was found among the studies (I^2^ = 22%); therefore, the data were analyzed using a fixed-effects model. The meta-analysis of these data demonstrated that the degree of postoperative IOL misalignment was significantly lower in the digital marking group compared to the manual marking group (WMD = −1.12, 95% CI: −1.68 to −0.55, *P* = 0.0001) (Fig. 7).

### Publication bias

**Fig. 8:**
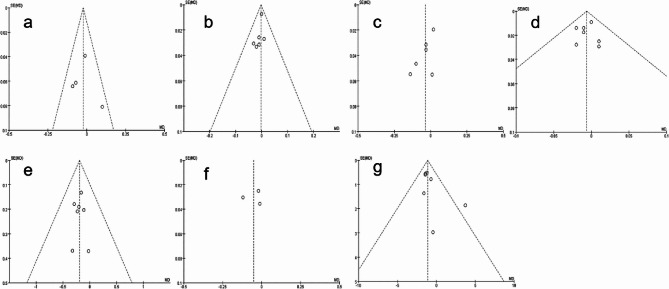
Funnel plots of the included studies for evaluating publication bias (**a**) preoperative UDVA (**b**) postoperative UDVA (**c**) preoperative CDVA (**d**) postoperative CDVA (**e**) preoperative astigmatism (**f**) mean deviation from TIA (**g**) postoperative IOL misalignment

The funnel plots depicting the preoperative UDVA, postoperative UDVA, preoperative CDVA, postoperative CDVA, preoperative astigmatism, mean deviation from TIA, and postoperative IOL misalignment exhibited symmetrical distributions, suggesting a low possibility of significant publication bias (Fig. 8).

## Discussion

Corneal astigmatism is one of the factors leading to reduced visual acuity following cataract surgery. Implanting a toric IOL during surgery can improve postoperative visual acuity and quality of life of patients. A crucial aspect in the application of toric IOLs is ensuring that the axial positioning of the intraocular lens precisely aligns with the intended target axis, as any deviation from this axis can result in the undercorrection of astigmatism or the occurrence of astigmatic rotation [17]. Accurate preoperative marking of the target axis is crucial for successful astigmatism correction. Previously, the most commonly used method for corneal marking was manual marking. Digital marking methods have emerged with advancements in technological equipment [18]. Currently, there is no consensus on whether the accuracy of digital marking is better than that of manual marking. The findings of this study indicated that the degree of postoperative IOL misalignment was significantly lower in the digital marking group than in the manual marking group. A possible explanation for this observation could be that during manual marking, slight eye movements by the patient may introduce displacement deviations as the doctor employs a marking pen to designate points on the horizontal axis of the corneal limbus. Conversely, the digital marking approach relies on the anterior segment image to ascertain both the horizontal axis and the target axis, enabling an accurate determination of the axis position even amidst eye movements, thus, the axis marking becomes more accurate, leading to reduced errors [19].

This study revealed no significant differences in UDVA or CDVA between the digital and manual marking groups at the 3-month postoperative assessment. This underscores the feasibility of both marking methods in clinical settings, particularly in healthcare facilities with limited financial resources that may not be able to invest in digital marking equipment. The manual marking method did not significantly compromise postoperative visual acuity recovery in patients. However, examination of the forest plot suggests that the digital marking method tended to exhibit a more favorable trend regarding improving postoperative visual function. Most studies showed that the postoperative visual acuity for eyes that underwent the digital marking method was better than that for the manual marking method, although the difference was not significant [8, 10, 13, 15]. Hence, more precise and less prone-to-error marking of the astigmatism axis resulted in reduced postoperative residual astigmatism, less misalignment of the intraocular lens (IOL), and ultimately improved visual recovery for patients after surgery [9, 20]. Hospitals with sufficient financial resources should adopt the digital marking method during IOL implantation surgery.

Our meta-analysis also revealed significant variations in preoperative astigmatism between the manual and digital marking groups; the digital marking group exhibited a higher degree of preoperative astigmatism than the manual marking group. Additionally, the findings of this study demonstrated that there was no significant difference in the mean deviation from the TIA between the two groups. This suggests that regardless of high preoperative astigmatism in a patient, a satisfactory TIA can still be achieved by using of an appropriate formula for calculating the precise power and axis of the toric IOL. The measurement of preoperative ocular biometric parameters is also crucial, including axial length, keratometry, anterior chamber depth, and posterior corneal astigmatism, which are all related to the accurate calculation and selection of toric IOL power [21]. Accurate determination of the target astigmatism axis is a pivotal factor that influences the level of postoperative residual astigmatism. For the effective correction of astigmatism, it is crucial to position the toric IOL precisely along its designated axis. Any deviation from the intended axis, either stemming from imprecise placement or unanticipated postoperative rotation within the capsular bag, can undermine the correction effectiveness. For instance, a 3-degree angular deviation can result in a nearly 10% reduction in astigmatism correction, whereas a 10-degree deviation can lead to up to 30% undercorrection [15, 22]. Consequently, the preoperative marking process is a crucial step in ensuring the desired outcome as it plays a decisive role in guiding the precise alignment of the toric IOL. However, this study confirmed that the digital marking technique resulted in a lower postoperative IOL misalignment rate than that of the manual marking technique; the WMD for IOL misalignment (−1.12 degrees) is statistically significant but probably clinically insignificant. A 3-degree misalignment causes an approximate 10% undercorrection, whereas a 1.12-degree misalignment results in approximately 3.7%. For a 7.0-diopter correction, this amounts to approximately 0.25-diopters, which is the minimal step of the refraction measurement. Clinically, it is quite uncommon to encounter patients with cataract and 7.0 diopters of astigmatism prior to surgery. In general, toric IOL rotations less than 10 degrees change an eye’s refraction less than 0.50 diopters in clinical practice [22]. Such slight axis rotations are not an obstacle for satisfactory astigmatism correction with toric IOLs. However, if the toric IOL rotates more than 10 degrees from the target axis, toric IOL realignment is required [23]. Ten degrees may be considered the minimal clinically important difference threshold for IOL misalignment. Although a statistically significant difference may not have clinical significance, the digital marking technique represents the pursuit of excellence for achieving more precise astigmatism correction.

Accurate preoperative marking is essential to minimize the risk of toric IOL misalignment. This manual marking process requires patients to sit upright in front of a slit-lamp microscope to ensure precision during the marking procedure. Typically, the preoperative stage commences with the establishment of a horizontal meridian spanning 0–180° as a reference [24]. During the surgical procedure, a secondary instrument such as a Mendez ring is employed to determine the targeted axis for the toric IOL, relying on the horizontal meridian [25]. However, manual marking can be intricate and requires a degree of proficiency in practice and experience. Using thick marker pens may result in wide or vague markings that are susceptible to fading or disappearing during surgery. Moreover, if the line connecting these marks deviates from the corneal center, additional imprecision can be introduced into the exact alignment of the toric IOL. Furthermore, manual marking is an invasive process that requires more time from surgeons, and its accuracy relies heavily on a high level of proficiency. As surgeons gain more experience in manual marking, they are likely to achieve more precise toric IOL alignment outcomes.

Digital marking adopts a noncontact methodology, eliminating any direct contact with patients’ eyes throughout the procedure. This method guarantees accurate markings, while minimizing psychological stress and ocular discomfort for the patients. Furthermore, it eliminates errors commonly associated with traditional ink markers. This methodology streamlines the marking process, boosts efficiency, and enables surgeons to complete tasks in a shorter timeframe [18, 26]. Nevertheless, digital image-guided marking relies on preoperative high-resolution anterior segment images, and it is imperative to avoid conjunctival edema or bleeding during surgery to maintain a clear operative field [27]. Failure to do so can hinder the execution of axis markings and potentially lead to misalignments. Although both marking methods have their respective merits and limitations, the findings of this study revealed that both techniques yielded comparable postoperative visual acuity outcomes, indicating their necessity and the inadvisability of eliminating manual marking. Hospitals should select the method that best aligns their capabilities and expertise.

This study had certain noteworthy limitations. First, our meta-analysis only included four RCTs; the remaining three studies featured retrospective designs. Given that the number of studies in each subgroup was too small and the data was scarce, we did not conduct a separate subgroup analysis for research design type to clarify the differences in results among different design studies. However, we conducted heterogeneity tests for each outcome parameter and selected different effect models based on the heterogeneity results for data analyses. Second, since only seven studies were included in this meta-analysis, the power of the funnel plot to detect publication bias was low. Despite this limitation, we still constructed a funnel plot to provide a visual reference, aimed to preliminarily observe the study distribution trend rather than serve as definitive evidence of publication bias. Third, this study did not have language restrictions to avoid language bias and gray literature (such as unpublished dissertations and research reports) was not included after team discussion due to the difficulty in verifying data integrity and reproducibility. This limitation restricted our ability to fully evaluate the potential skewing of the evidence base due to unpublished or underreported studies. Fourth, a sensitivity analysis was not performed in this study, mainly due to the limited number of included studies, which was only seven. When the number of studies is small, excluding any single study may significantly alter the overall effect size, leading to considerable instability or misleading interpretations of the results. Given this constraint, we chose to explicitly state this limitation rather than conduct an analysis with questionable reliability. Finally, parameters such as total surgical duration, surgically induced astigmatism, post-traumatic astigmatism, different toric IOL types, surgeon skill, and surgical settings are essential for assessing the efficacy of both marking techniques. Unfortunately, an analysis of these parameters was not performed because of the lack of sufficient data to comprehensively examine them. Therefore, future research should prioritize conducting large-scale, longitudinal, and rigorously designed RCTs to thoroughly evaluate these parameters and gain deeper insights into the potential differences between the two marking methods.

## Conclusions

The results of this meta-analysis indicated a notable reduction in the degree of postoperative IOL misalignment in patients who underwent digital marking compared with those who underwent manual marking. However, there were no significant differences in postoperative UDVA and CDVA between the two groups. Both marking methods are encouraged in clinical practice due to their effectiveness and accuracy; although, digital marking is highly recommended owing to its lower IOL misalignment rate. To evaluate the precision of these marking techniques accurately, further research with a larger sample size and long-term follow-up is warranted.

## Supplementary Information

Supplementary Material 1.

## Data Availability

No datasets were generated or analysed during the current study.

## Abbreviations

IOL: Intraocular lens
UDVA: Uncorrected distance visual acuity
CDVA: Corrected distance visual acuity
log MAR: Logarithm of minimal angle of resolution
RCTs: Randomized controlled trials
WMD: Weighted mean difference
Cls: Confidence intervals
TIA: Target-induced astigmatism
Retro: Retrospective study
CES: Callisto Eye System
DM: Digital marking
MM: Manual marking
NR: Not reported
